# Phrenic nerve stimulation for treatment of central sleep apnea

**DOI:** 10.3389/frsle.2023.1214363

**Published:** 2023-07-18

**Authors:** Shahrokh Javaheri, Robin Elizabeth Germany, Kara Dupuy-McCauley

**Affiliations:** ^1^Bethesda Medical Center, Cincinnati, OH, United States; ^2^Division of Cardiovascular Disease, The Ohio State University, Columbus, OH, United States; ^3^Division of Pulmonary, Critial Care and Sleep Medicine, University of Cincinnati, Cincinnati, OH, United States; ^4^Division of Cardiovascular Diseases, University of Oklahoma Health Sciences Center, Oklahoma City, OK, United States; ^5^ZOLL Respicardia, Inc, Minnetonka, MN, United States; ^6^Center for Sleep Medicine, Mayo Clinic, Rochester, MN, United States

**Keywords:** central sleep apnea, neurostimulation, sleep disordered breathing, phrenic nerve, cardiovascular disease, heart failure

## Abstract

The prevalence of central sleep apnea (CSA) is rare in general population. However, CSA is prevalent in those with cardiovascular and cerebrovascular disorders. CSA may persist or even worsen with positive airway pressure therapy in some patients and phrenic nerve stimulation (PNS) offers an alternative treatment for patients with CSA. The device is implanted similar to a cardiac pacemaker and typically followed in the sleep clinic. Multiple studies have described the efficacy and safety of PNS. Improvements were seen in apnea hypopnea events, central events, arousals, and daytime sleepiness and maintained through 5 years. Safety demonstrated a 91% freedom from serious adverse events through 1 year. The physiologic approach and improvement in sleep metrics and quality of life with a strong safety profile make this therapy a good option for many patients with central sleep apnea.

## Introduction

Compared to obstructive sleep apnea (OSA), the prevalence of central sleep apnea (CSA) is rare in general population. Similarly, while there are many treatment options today for OSA, treatment options for CSA are few. Phrenic nerve stimulation (PNS) represents one of the newest treatment options for patients with CSA. The device is implanted by a cardiologist in the cardiac suite and programmed by sleep specialists. Clinical data demonstrates improvement in sleep metrics with safety similar to other neurostimulation systems. It is important for sleep clinicians today to understand where the benefit and risk of this therapy for this unique patient population.

**Figure 1:**
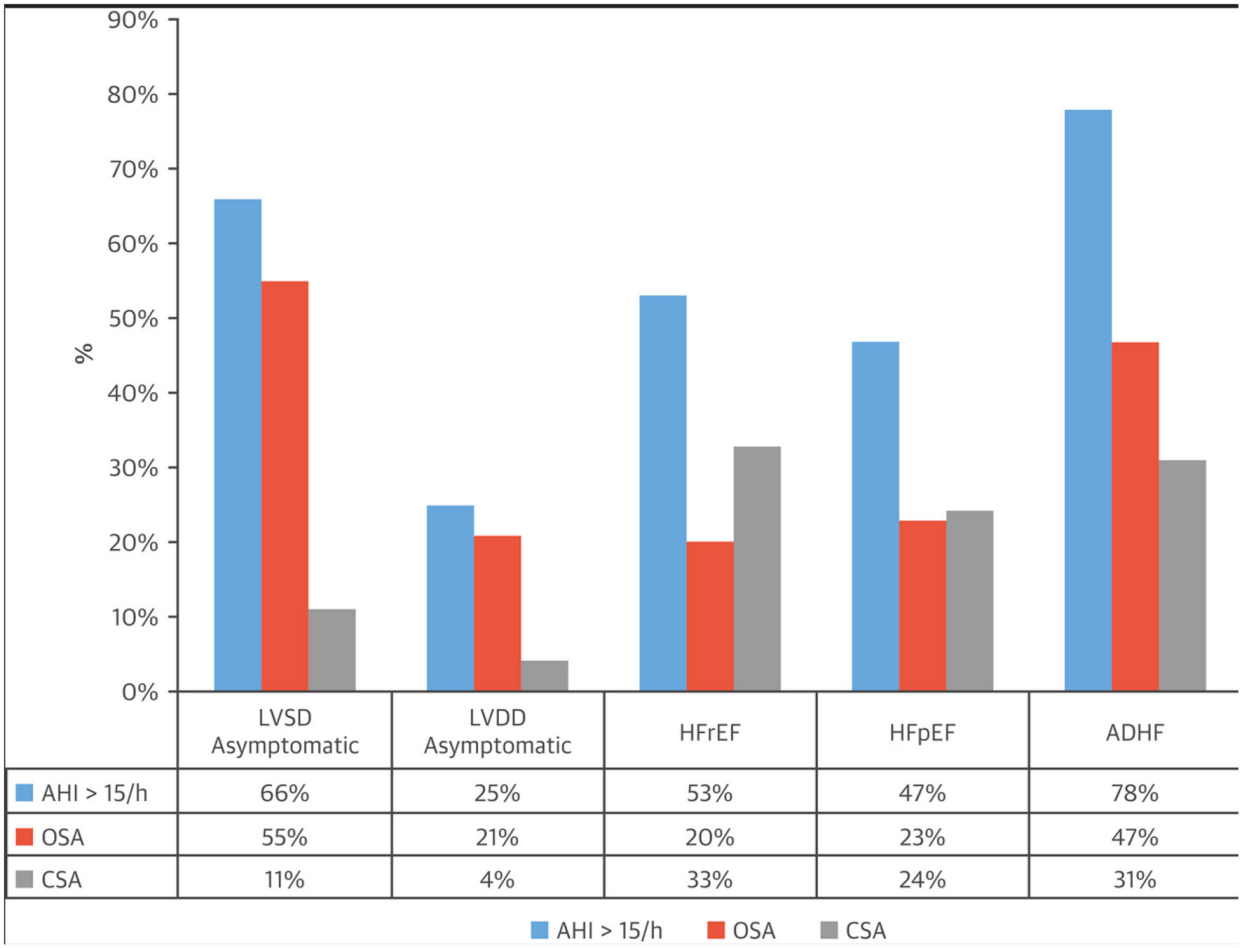
Prevalence of central sleep apnea in heart failure.

In adults, CSA is prevalent in certain conditions (Javaheri and Badr, 2023), most commonly in those with cardiovascular and cerebrovascular disorders (Javaheri et al., 2017). Among cardiac disorders, left ventricular systolic dysfunction, heart failure with reduced ejection (HFrEF) is the most common (Figure 1). However, CSA can also be comorbid with symptomatic and asymptomatic left ventricular dysfunction (Lanfranchi et al., 2003), and atrial fibrillation (Sin et al., 1999). The association with CSA has been best documented in AF in association with HFrEF. In one study of 100 patients with HFrEF, 80% of those with AF had CSA (Javaheri et al., 1998). Importantly, in a long-term prospective study of 2,865 community-dwelling older men who underwent a baseline polysomnogram (PSG) and were followed for a mean 7.3 years, elevated central apnea index (CAI) and Hunter Cheyne-Stokes Breathing (HCSB) was significantly associated with increased risk of decompensated heart failure and/or development of clinical heart failure (Javaheri et al., 2016). Atrial fibrillation is associated with CSA not only in those with reduced ejection fraction, but also with those with preserved ejection fraction (Bitter et al., 2009).

### Phrenic nerve stimulation for treatment of CSA

Whereas, continuous positive airway pressure (CPAP) is quite effective in the treatment of OSA, it is ineffective in a large number of subjects with CSA such those with heart failure (Javaheri and McKane, 2020) or those on opioids (Javaheri et al., 2014). In these individuals, CSA persists or may worsen with positive airway pressure therapy, whereas phrenic nerve stimulation (PNS) is quite effective in virtually eliminating central sleep apnea.

Following a pivotal randomized control trial (Costanzo et al., 2016) in 2017, the FDA approved a transvenous phrenic nerve stimulation (TPNS) device (remedē system, ZOLL Medical, Minnetonka, MN) for the treatment of CSA of various causes.

Historically, it is interesting to note that although PNS was approved by the FDA in 2017, this is not a new idea. In Sarnoff et al. (1948) demonstrated for the first time that artificial respiration could be effectively administered to the cat, dog, monkey, and rabbit in the absence of spontaneous respiration by electrical stimulation of one (or both) phrenic nerves (Sarnoff et al., 1948). In later experiments, these investigators showed that unilateral phrenic nerve stimulation is also equally effective in humans as they had exhibited in animal models (Wittenberger et al., 1949).

### The system and the algorithm

**Figure 2:**
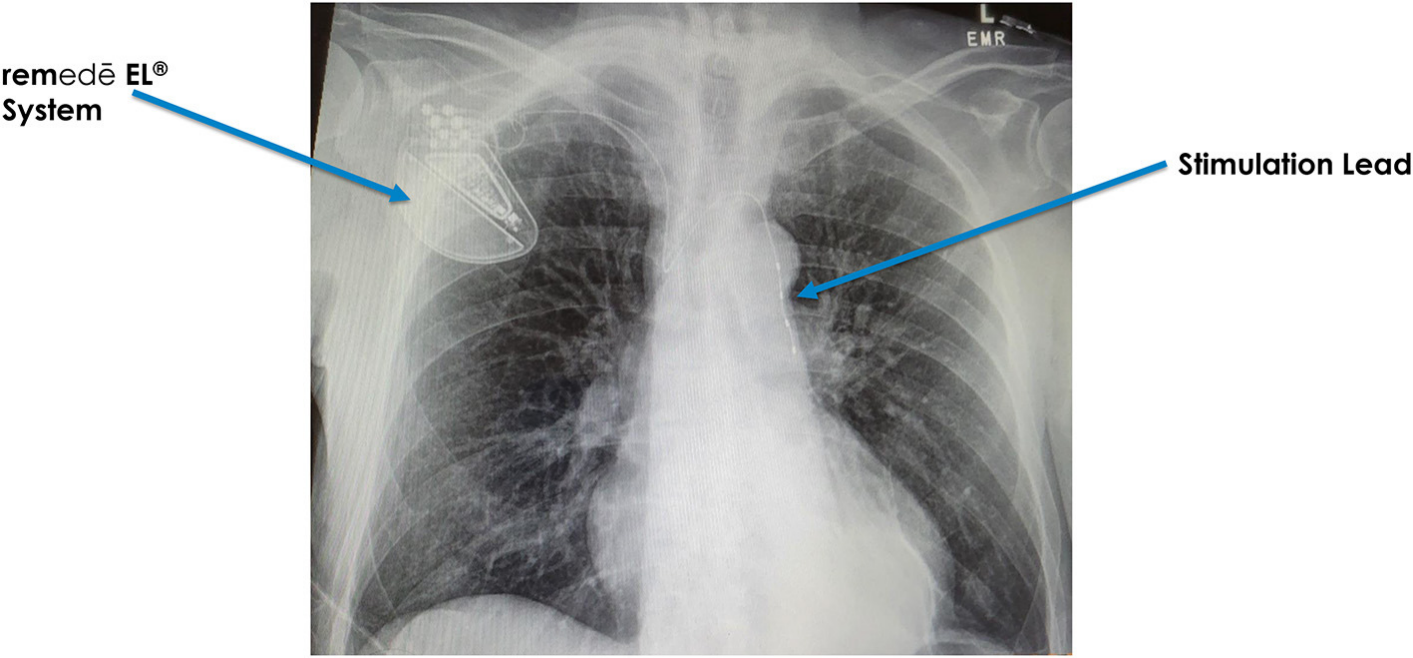
Transvenous phrenic nerve stimulation system.

The phrenic nerves pass over and come in close proximity with veins, both on the right (brachiocephalic) and on the left (pericardiophrenic vein) (Figure 2). Similar to cardiac pacemaker implantation, an electrophysiologist places the stimulation lead within the vein in close proximity to the phrenic nerve. The stimulation lead is typically introduced on the right side below the clavicle and then attached to the pulse generator, which is placed under the skin in the right pectoral area (Augostini et al., 2019). The procedure typically lasts between 2 and 3 h, is completed under conscious sedation and patients typically go home the same day.

**Figure 3:**
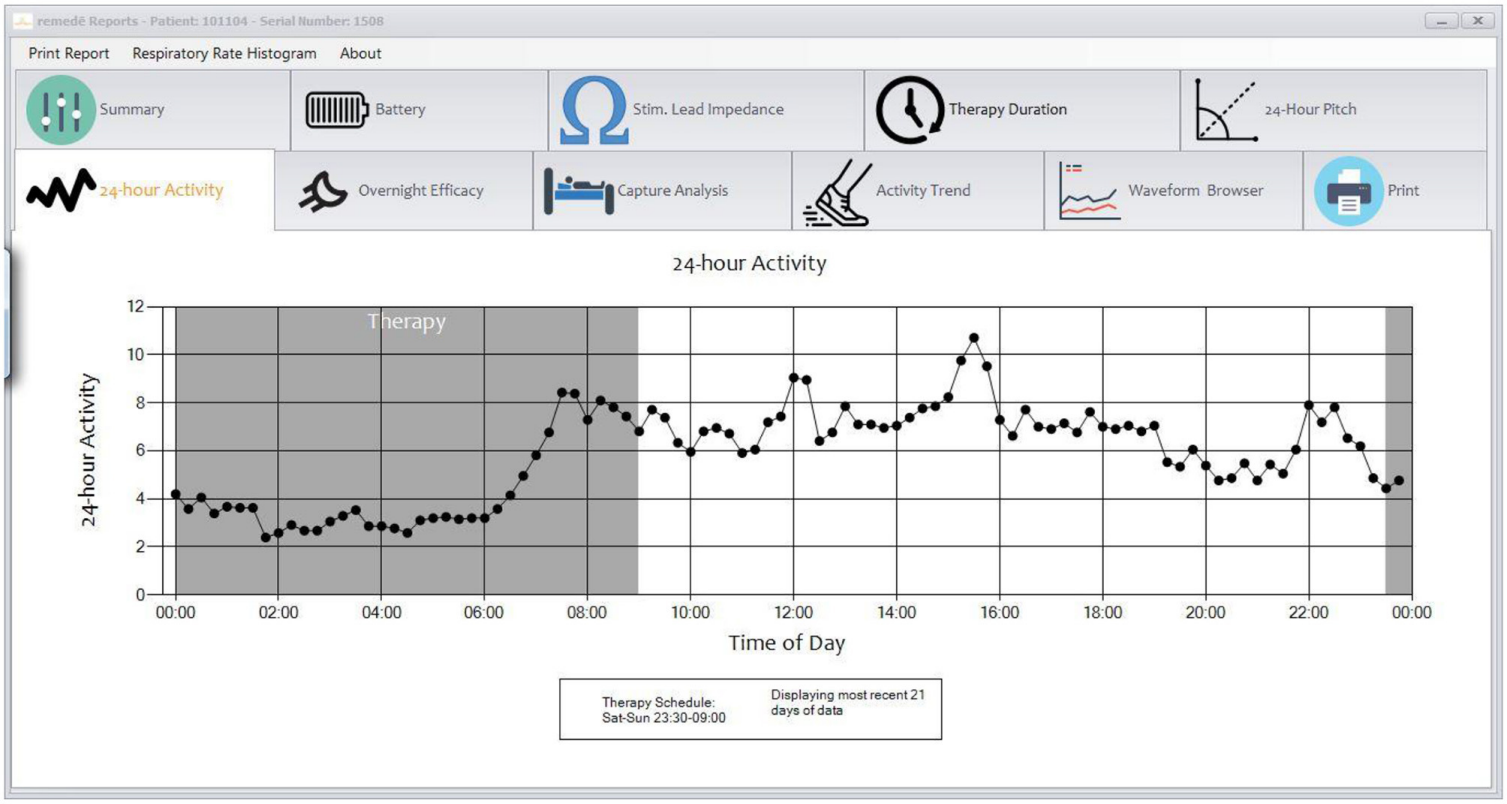
Sample diagnostic information from the remedē^®^ system.

**Figure 4:**
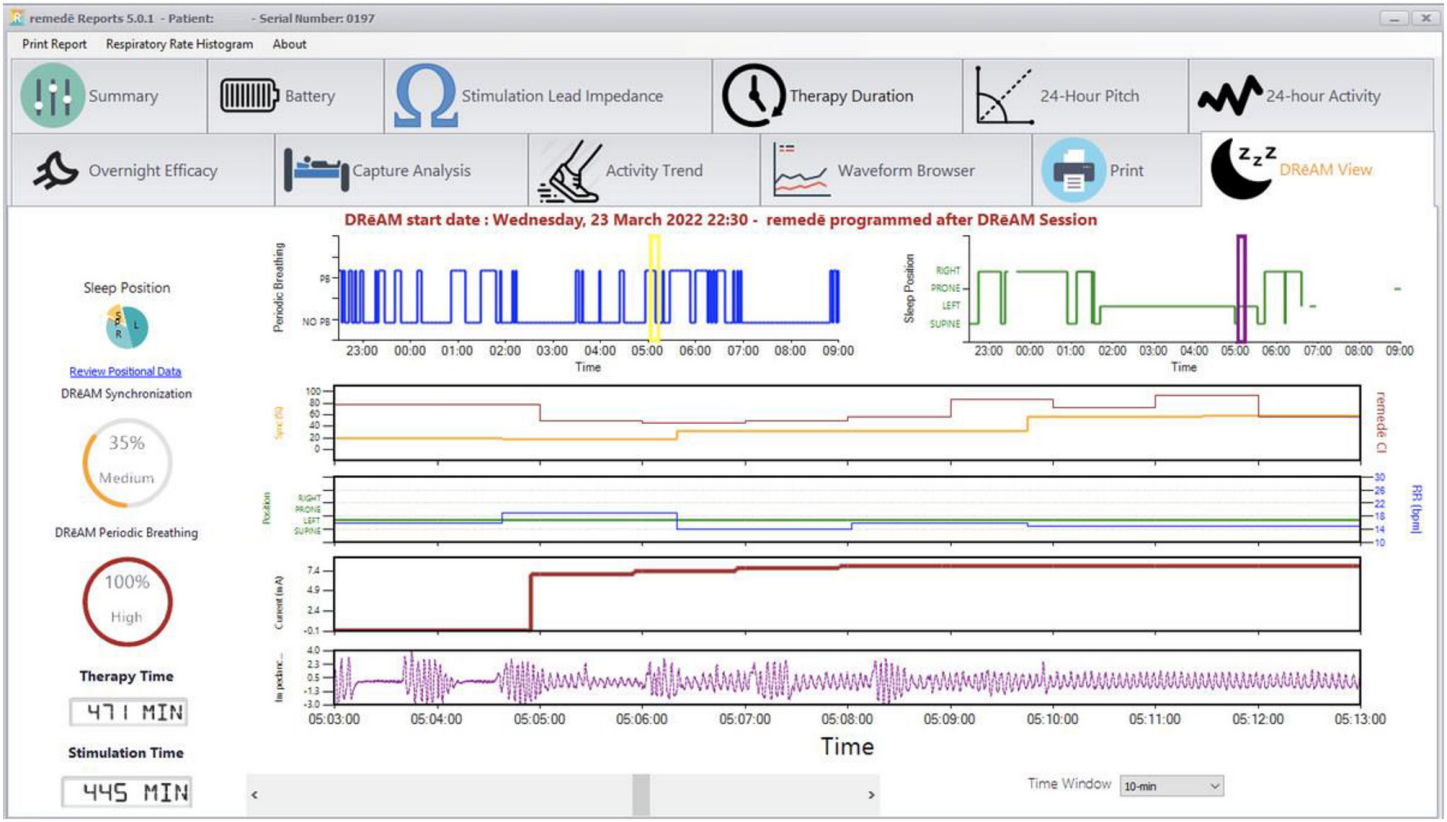
Sample diagnostic information: DRēAM view screen.

**Figure 5:**
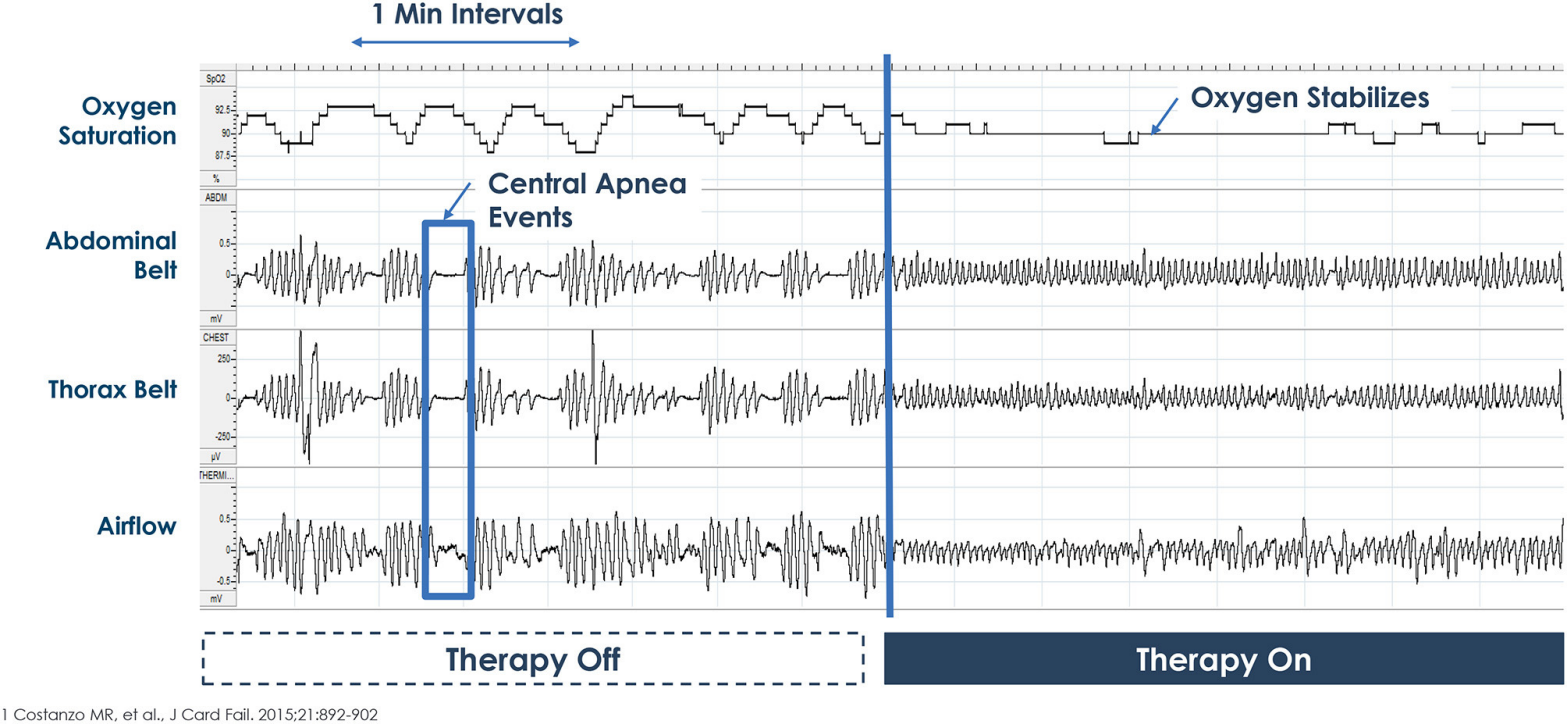
Example of therapy Off-On during overnight polysomnogram. Picture courtesy ZOLL Respicardia: This is an individual patient example and does not provide any indication, guide, warranty or guarantee as to the response other patients may have to the therapy. Individual results may vary.

The therapy is activated in the sleep medicine clinic ~6 weeks after implantation using a programmer similar to a tablet computer. The device collects information regarding position, breathing and activity at night and this information can be used to program the device (Figures 3, 4). Following the initial programming session, therapy is personalized over the next few months and then efficacy is confirmed with a sleep study (Figure 5). Typically, the device is programmed to lower the respiratory rate with a slightly longer and deeper breath.

This is in contrast to other diaphragmatic or phrenic nerve stimulation systems which increase both respiratory rate and tidal volumes. These systems are typically placed in the operating room under general anesthesia with electrodes placed touching the phrenic nerve. These systems are often bilateral and the batteries are external and rechargeable. The tidal volume and rates can be changed by the patient or family similar to a ventilator and they are designed to take the place of mechanical ventilation in the case of spinal cord injury or central congenital hypoventilation syndrome (Headley et al., 2021). In other words, they are designed to increase minute ventilation whereas the transvenous PNS (remede system) is designed to stabilize breathing (stabilize carbon dioxide) and indicated for CSA in adults (Schwartz et al., 2020).

### Clinical studies

Multiple studies have described the efficacy and safety of PNS.

#### Feasibility and pilot studies

A proof-of-concept study (Ponikowski et al., 2012) was completed in sixteen patients with CSA. Overnight unilateral stimulation of phrenic nerve resulted in virtual elimination of CAI (27 to 1 events/hour of sleep, *P* ≤ 0.001). There was also significant reduction in the apnea hypopnea index (AHI) with the median decreasing from 45 to 23 events/ hour of sleep (*P* = 0.002). There were no significant changes in obstructive apnea index (OAI); the residual events were primarily hypopneas. In concert with reduction in CAI, similar changes occurred in arousal index (32 to 12 events/hour of sleep, *P* = 0.001]. Oxygen desaturation index of 4% (ODI4%) decreased from 31 to 14 events/hour of sleep, *P* = 0.002]. The feasibility study was followed by a pilot study (Abraham et al., 2015) which demonstrated chronic efficacy at 3 months with a reduction in AHI from baseline of 49.5 +/- 14.6 events per hour of sleep to 22.4 ± 13.6 events per hour of sleep; *p* < 0.0001) with follow-up through 1 year (Jagielski et al., 2016). Additionally, improvements were noted in sleepiness (Epworth Sleepiness Scale improved 4.1 points from baseline) and quality of life compared to baseline (76% noted improvement in health) (Abraham et al., 2015).

### Pivotal trial

In this trial (Costanzo et al., 2016), 151 eligible patients with moderate or severe CSAwere randomly assigned to the treatment (*n* = 73) or control (*n* = 78) groups at the time of implantation. Participants in the active arm received PNS for the next 6 months. All PSG were centrally and blindly scored. There were significant decreases in AHI, CAI, arousal index, % time in rapid eye movement (REM) sleep and ODI4% (Table 1). The difference between the treatment and control group demonstrated a 25 event/hour reduction in AHI and 23 event/hour reduction in CAI. Importantly, daytime sleepiness and patient global assessment were statistically improved compared to the control group. Following the six-month randomization period, all patients had therapy activated and were followed until the end of the trial at ~3 years.

**Table 1:** Differences between treatment and control group in the remedē^®^ system pivotal trial (6 months data).

	**Between group change (treatment versus control) *n =* 132 (change +/– standard deviation)**	
Central apnea index (CAI)	−23 +/– 18	*P* < 0.0001
Apnea hypopnea index (AHI)	−25 +/– 18	*P* < 0.0001
Arousal index (AI)	−15 +/– 19	*P* < 0.0001
Percent of sleep in REM sleep	2 +/– 8	*P* = 0.024
Moderate or marked improvement in patient global assessment	55 (40–68)	*P* < 0.0001
Oxygen desaturation index (ODI) 4%	−23 +/– 18	*P* < 0.0001
Epworth Sleepiness Scale (ESS)	−3.7 +/– 5.0	*P* < 0.0001

In general, CSA is less prevalent in REM sleep than in non-REM (NREM) sleep (Orr et al., 2016). In the pivotal trial, the CAIs in NREM and REM sleep were 28 and 8/h of sleep, respectively. In order to determine the efficacy of PNS to improve CSA during REM sleep, we performed a separate assessment of patients from the pivotal trial. We compared changes in sleep apnea indices from baseline to 6 months in REM and NREM sleep for treatment (active TPNS therapy, *n* = 50) and control (inactive device, *n* = 57). The analysis was performed only in patients who had at least 5 min of REM sleep in both the initial and follow up PSG. Similar to findings from the pivotal trial, we found the AHI decreased significantly during both REM and NREM sleep in patients with TPNS. Compared to baseline, the mean REM AHI decreased significantly from 28/h of REM sleep to 8/h in the active arm. The respective values in the control group were 20/h of REM at baseline and 25/h at 6 month follow up.

Also, similar to the data in the pivotal trial, the reduction in AHI was driven by reductions in central events. Compared to baseline, respective median values for REM CAI were 8 at baseline and 0 with treatment at 6 months.

This analysis suggests that although CSA is traditionally associated with NREM sleep, patients with CSA have a significant, albeit lower, number of centrally mediated disordered breathing events in REM sleep, and PNS improves CSA in both REM and NREM sleep.

### Long term studies

Efficacy and safety through 12 months (Costanzo et al., 2018b) were reported. Similar improvements were demonstrated in the control group and the initial treatment group once activated including improvements in AHI, CAI, arousal index and oxygenation. Patient global assessment demonstrated a similar improvement in overall quality of life in 74% and a moderate or marked improvement in overall health in 58% of the former control group once therapy was activated, similar to the treatment group, at 6 months.

Additional long-term data was gathered in a subset of patients enrolled in a post-approval study through 5 years (Costanzo et al., 2022). Patients underwent an in-lab attended PSG at 5 years. Improvements in sleep metrics continued through the 5 years of the study as well as improvements in daytime sleepiness and included a 22 event per hour reduction in AHI with a median CAI of 1 event/hour (95% CI 0.5).

One additional investigator-initiated trial was completed by Fox et al. (2019) and demonstrated a similar safety and efficacy profile to the pivotal study. All patients enrolled had heart failure with reduced ejection fraction. AHI improved from 38 +/– 18 to 17 +/– 9 (*P* = 0.01) and time below 90% improved from 81 +/- 56 min to 28 +/– 43 min (*P* < 0.01). While no improvement in ejection fraction was noted, there was a 40-meter improvement in 6-minute hall walk test (*P* = 0.035).

## Heart failure

There is particular interest of the treatment of CSA in patients with heart failure following the surprising results of the SERVE-HF study, which demonstrated increases in cardiovascular mortality with the treatment of CSA (Cowie et al., 2015). The subset of patients with HF was evaluated in a study by Costanzo et al. (2018a). This group was 64% of the overall study group in the Pivotal Study and had similar improvements in sleep metrics. In addition, an improvement in disease-specific quality of life was seen in the Minnesota Living with Heart Failure scale −6.8 ± 20.0 (*P* = 0.005) at 1 year compared to baseline (Costanzo et al., 2018a). There was a small improvement in left ventricular ejection fraction of 4.0% (interquartile range −1.0 to 8.0%; *P* = 0.004) and a positive trend in time to first heart failure hospitalization with rates of 4.7% (standard error = 3.3) in the treatment group and 17.0% (standard error = 5.5) in the control group (*P* = 0.065). There was no difference between the treatment and control group in mortality, but there was only 6 months of randomized data.

### Idiopathic central sleep apnea

ICSA is a relatively rare disorder. Patients may present with insomnia, daytime fatigue, and sleepiness. In a small sub-study (Javaheri et al., 2020) of 16 patients with moderate to severe central sleep apnea (baseline AHI = 40, CAI = 25), PNS improved at 6, 12, and 18 months of therapy: the AHI decreased by 25, 25, and 23 events/h (*P* < 0.001 at each visit) and the central apnea index by 22, 23, and 22 events/h (*P* < 0.001 at each visit). Furthermore, the arousal index decreased by 12 (*P* = 0.005), 11 (*P* = 0.035), and 13 events/h (*P* < 0.001). Quality of life instruments showed clinically meaningful improvements in daytime somnolence, fatigue, general and mental health, and social functioning. The only related serious adverse event was lead component failure in one patient.

## Safety of phrenic nerve stimulation

Safety of phrenic nerve stimulation has been studied with different systems for over 50 years (Sarnoff et al., 1948). Initial studies on the transvenous system found safety similar to other neurostimulation platforms, but with some lead issues related to dislodgement with the initial lead design (Abraham et al., 2015). The lead was redesigned for the pivotal trial to maintain better stability over time.

In the pivotal trail, 138 (91%) of 151 patients had no serious related adverse events at 12 months. Seven (9%) cases of related-serious adverse events occurred in the control group and six (8%) cases were reported in the treatment group (Table 2). Seven patients died (unrelated to implant, system, or therapy), four deaths (two in treatment group and two in control group) during the 6-month randomization period when neurostimulation was delivered to only the treatment group and was off in the control group, and three deaths between 6 months and 12 months of follow-up when all patients received neurostimulation. Twenty-seven (37%) of 73 patients in the treatment group reported non-serious therapy-related discomfort that was resolved with simple system reprogramming in 26 (36%) patients but was unresolved in one (1%) patient. Complications between year 1 and 5 occurred in 5% of patients and were primarily related to lead issues. However, there were three episodes in two patients of interactions with cardiac devices. These resolved with reprogramming, but physicians should be aware of the possibility of interaction.

**Table 2:** Serious adverse events with the remedē^®^ system through 12 months in the pivotal trial.

**Serious adverse event**	**Number of patients (*N =* 151) (%)**
Impending pocket erosion	2 (1%)
Implant site hematoma	1 (1%)
Implant site infection	2 (3%)
Extra-respiratory stimulation	1 (1%)
Concomitant device interaction	1 (1%)
Lead component failure	1 (1%)
Lead dislodgement	2 (3%)
Lead displacement	1 (1%)
Non-cardiac chest pain	1 (1%)
Elevated transaminase	1 (1%)

### Advantage and disadvantages of PNS

In contrast to positive airway pressure devices which increase intrathoracic pressure and could result in adverse hemodynamic consequences, particularly in the setting of heart failure, PNS therapy is a physiological treatment mimicking normal breathing. Here we note that in the largest randomized clinical trial for the treatment of CSA, the SERVE-HF trial, there was a significant association with use of adaptive servo-ventilation (ASV) and cardiovascular mortality when compared to the control arm. The investigators hypothesized that one potential reason for this association was the increased intrathoracic pressure imposed by the device. PNS is devoid of this adverse side effect.

Another advantage of PNS is adherence to therapy. The therapy activates automatically at night as long as the patient is in a sleeping position. Notably, both in the CANPAP and the SERVE-HF trials, adherence to CPAP and ASV was about 3 to 4 h (Bradley et al., 2005; Cowie et al., 2015). Because, the burden of CSA increases in late hours of NREM sleep (Javaheri, 2000), full adherence to PNS provides additional benefit, compared to mask therapy. We note that residual hypopneas may remain after PNS. Changing the programming of the device over time can improve the number of events. If residual events are obstructive, low CPAP could be effective (Beyerbach et al., 2019).

## Clinical implications

**Figure 6:**
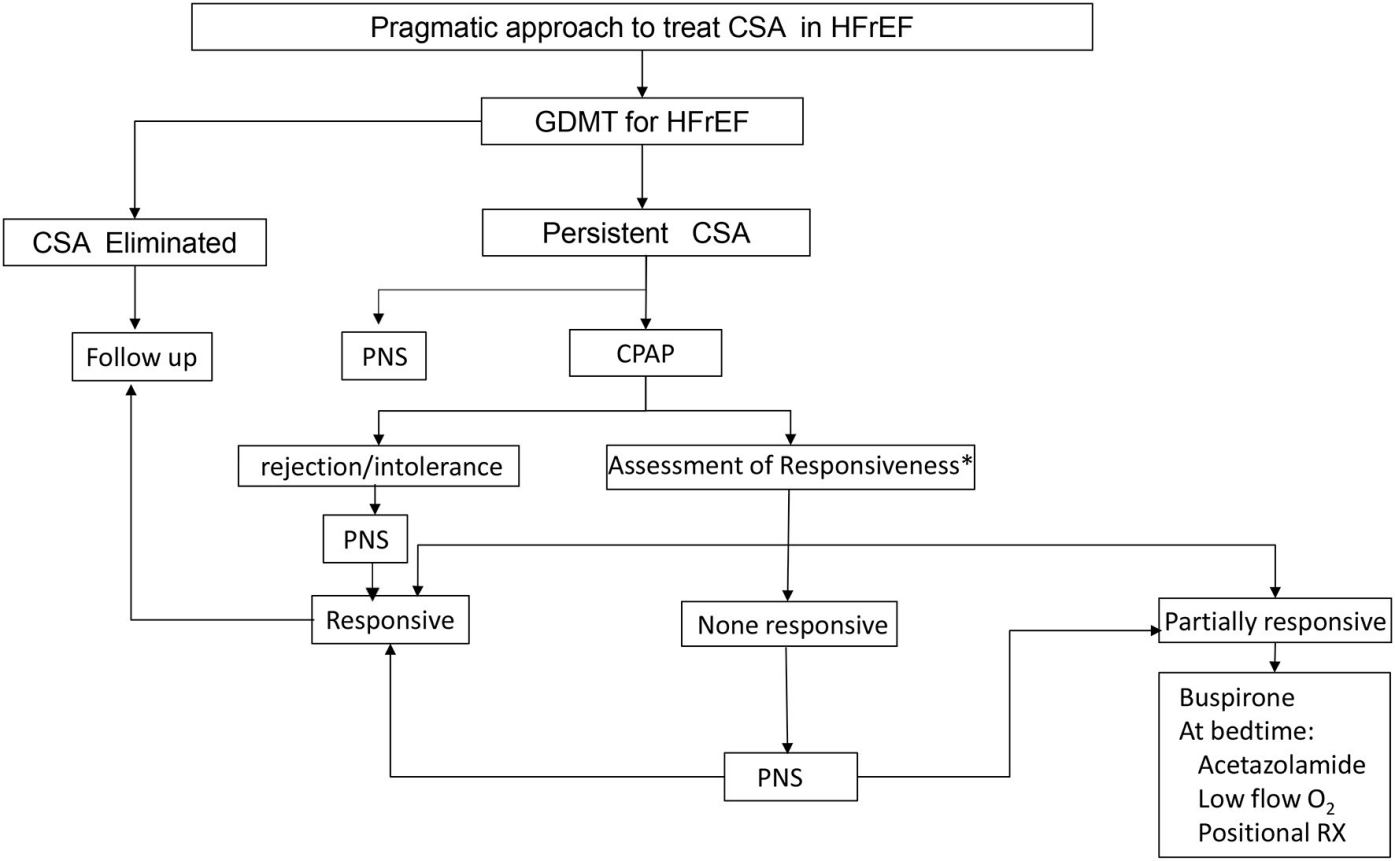
Proposed CSA treatment algorithm. Treatment should always start with guideline directed medical therapy (GDMT) and options for treatment include continuous positive airway pressure (CPAP), phrenic nerve stimulation (PNS), medications and oxygen.

TPNS is now available at more than one hundred centers in the United States. Determining which patients are most appropriate for this therapy takes both the sleep metrics and patient co-morbidities. Specifically, patients with low ejection fractions have few therapeutic options and may be early candidates for TPNS. We have previously proposed the following flowchart for treatment of CSA (Figure 6) (Javaheri et al., 2020). Once implanted, patients will have several visits in the sleep clinic and a follow up sleep study to optimize the programming of the device. Understanding this follow-up pathway will be important to the prescribing physician.

TPNS is an implantable device, and the cost is high, like hypoglossal nerve stimulation, compared to other sleep apnea therapies. Large scale, long-term studies related to mortality are not yet available. However, the physiologic approach and improvement in sleep metrics and quality of life with a strong safety profile make this therapy a good option for many patients with CSA.

## Author contributions

SJ was the lead author and developed the concept and outline. All authors contributed to the article and approved the submitted version.
